# Sonographic features of uterine fibroids that predict the ablation rate and efficacy of high-intensity focused ultrasound

**DOI:** 10.3389/fonc.2024.1451626

**Published:** 2024-08-16

**Authors:** Hailan Xue, Songsong Wu, Kunhong Xiao, Guisheng Ding, Sheng Chen

**Affiliations:** ^1^ Fujian Maternity and Child Health Hospital College of Clinical Medicine for Obstetrics and Gynecology and Pediatrics, Fujian Medical University, Fuzhou, China; ^2^ Shengli Clinical Medical College of Fujian Medical University, Fuzhou, China; ^3^ Department of Ophthalmology and Optometry, Fujian Medical University, Fuzhou, China; ^4^ Ultrosound Department, Fujian Medical University Union Hospital, Fuzhou, China

**Keywords:** uterine fibroids, sonographic features, high-intensity focused ultrasound, ablation rate, energy efficiency factor

## Abstract

**Objective:**

This study aimed to identify the sonographic indicators that predict the ablation rate and efficiency of uterine fibroids during high-intensity focused ultrasound (HIFU) treatment.

**Methods:**

In this retrospective study, we analyzed the clinical data of patients with uterine fibroids who underwent HIFU treatment at Fujian Provincial Hospital between April 2019 and April 2022. Routine abdominal ultrasound examinations were performed to observe potential indicators before the HIFU treatment. After the treatment, enhanced magnetic resonance imaging (MRI) examination was performed within 2 weeks. The fibroid and non-perfused volumes (NPV) were determined, and the ablation rate and energy efficiency factor (EEF) were calculated.

**Results:**

A total of 75 patients (124 uterine fibroids) were included in this study. Uterine fibroids with a larger volume, high echogenicity, elliptical/diffuse leaf shape, and a posterior attenuation band had a higher HIFU ablation rate (*p*<0.05). Uterine fibroids with a larger volume and high echogenicity and without necrotic areas had a lower EEF (*p*<0.05). Multiple comparisons between fibroid types revealed statistically significant differences in EEF between subserosal and submucosal fibroids *(p* < 0.05) and between subserosal and mixed-type fibroids (*p* < 0.05). However, no statistically significant difference was observed between mixed-type and submucosal fibroids. The HIFU ablation rate and EEF showed no significant differences based on location within the wall and blood flow within the fibroids.

**Conclusion:**

Sonographic features of uterine fibroids can predict the rate and efficiency of HIFU ablation, providing useful guidance in selecting appropriate treatment for patients.

## 1 Introduction

Uterine fibroids, the most common benign tumors of the female reproductive system (1), affect 25-40% of women of childbearing age (2). While many patients are asymptomatic, others experience severe symptoms, such as heavy menstrual bleeding, prolonged menstruation, and pelvic pain. Other symptoms may be related to the compression caused by the mass, including frequent urination, urgency, constipation, and a sensation of pelvic pressure. Larger fibroids may also negatively affect fertility. Treatment is necessary for clinically symptomatic uterine fibroids. However, fibroids often regrow to their pre-treatment size even after intervention (3).

The definitive treatment for uterine fibroids is hysterectomy; however, it is unsuitable for patients who desire pregnancy. Myomectomy is an alternative treatment for those aiming to retain uterine function and fertility, but it is invasive and increases the risk of uterine rupture in subsequent pregnancies. High-intensity focused ultrasound (HIFU) ablation offers a non-invasive alternative with proven safety and efficacy for treating uterine fibroids (4–7). Nevertheless, some fibroids exhibit a suboptimal response to HIFU, emphasizing the need for an improved preoperative assessment to select suitable candidates. Magnetic resonance imaging (MRI) is an option for preoperative fibroid evaluation for response to HIFU (8–10). However, the preoperative use of MRI has limitations, including limited availability, prolonged examination time, high cost, and exclusion of specific patients. Therefore, a more economical, straightforward, and widely applicable preoperative test is crucial to guide the selection of uterine fibroids for HIFU treatment.

With advances in technology and the rapid development of medical devices, ultrasound has become increasingly sophisticated, offering excellent tissue contrast and image reproducibility. Ultrasound examinations are convenient, fast, accurate, safe, non-invasive, and highly reproducible. Consequently, ultrasound imaging is promising as a preoperative assessment of uterine fibroids for HIFU treatment. However, research investigating whether the sonographic features of fibroids can predict effective HIFU treatment is lacking. This study aimed to explore sonographic characteristics of uterine fibroids to identify parameters that can predict the ablation rate and efficiency of HIFU treatment.

## 2 Materials and methods

### 2.1 Study population

A retrospective analysis was conducted on the clinical data of 361 patients with uterine fibroids who underwent HIFU ablation at the ultrasound department of Fujian Provincial Hospital between April 2019 and April 2022.

### 2.2 Inclusion and exclusion criteria

The inclusion criteria were as follows: 1) patients who underwent ultrasound examination the day before or on the day of HIFU treatment and 2) patients who underwent follow-up enhanced MRI within 2 weeks of HIFU treatment. The exclusion criteria were as follows: 1) patients who underwent special interventions before or during HIFU treatment (e.g., local ethanol injection or transcatheter arterial embolization), 2) patients with pedunculated subserosal fibroids and fibroids in specific locations such as the cervix or broad ligament, and 3) patients with incomplete data.

This study was approved by the Ethics Committee of Fujian Provincial Hospital (Approval No: K2021-04-038). All participants signed standard informed consent forms during their medical treatment, agreeing that their data could be used for research purposes.

### 2.3 Instruments and equipment

The treatment utilized a JC200-focused ultrasound tumor therapy system (Chongqing Haifu Technology Co., Ltd.). This system performs real-time ultrasound positioning, HIFU three-dimensional scanning, and treatment. Controlled by a computer, it automatically locates the predetermined tumor target area, determines the treatment range, conducts three-dimensional conformal scanning, monitors, and analyzes the treatment effects in real-time, and provides feedback control for the treatment dosage. The main treatment parameters were as follows: 1) treatment frequency of 0.97 MHz; 2) focal width of 3 mm; 3) focal length of 8 mm; 4) focal distance of 162 mm; and 5) maximum power of 400 W for a single-point treatment.

For ultrasound examinations, the SIEMENS S3000 Color Doppler Ultrasound Diagnostic Instrument with a 6C1 convex array probe (frequency: 2.0–5.0 MHz) was employed. MRI examinations were conducted using Philips Achieva 1.5T and 3.0T MRI systems.

### 2.4 Examination methods, procedures, and treatment

#### 2.4.1 Ultrasound examination

On the day before or the day of the HIFU treatment, two-dimensional grayscale and color Doppler flow ultrasound imaging of the uterine fibroids were performed, and the images were stored. All patients underwent an ultrasound examination with an empty bladder. The examinations were conducted transabdominally with the patients in a supine position. The ultrasound was adjusted to achieve optimal resolution by scanning the pelvic cavity, uterus, and bilateral adnexal areas in longitudinal, transverse, and oblique sections. Fibroid location, size, morphology, number, internal echo characteristics, presence of necrotic areas, boundaries, attenuation zones, blood flow, and their relationship with the endometrium and surrounding organs was noted. Measurements of the three axes of the uterine fibroids were obtained from the maximum cross-sectional and vertical-sectional images. Subsequently, SonoVue ultrasound contrast was injected intravenously for contrast-enhanced ultrasound evaluation of fibroid perfusion.

#### 2.4.2 Observational parameters in ultrasound examination

**Figure 1:**
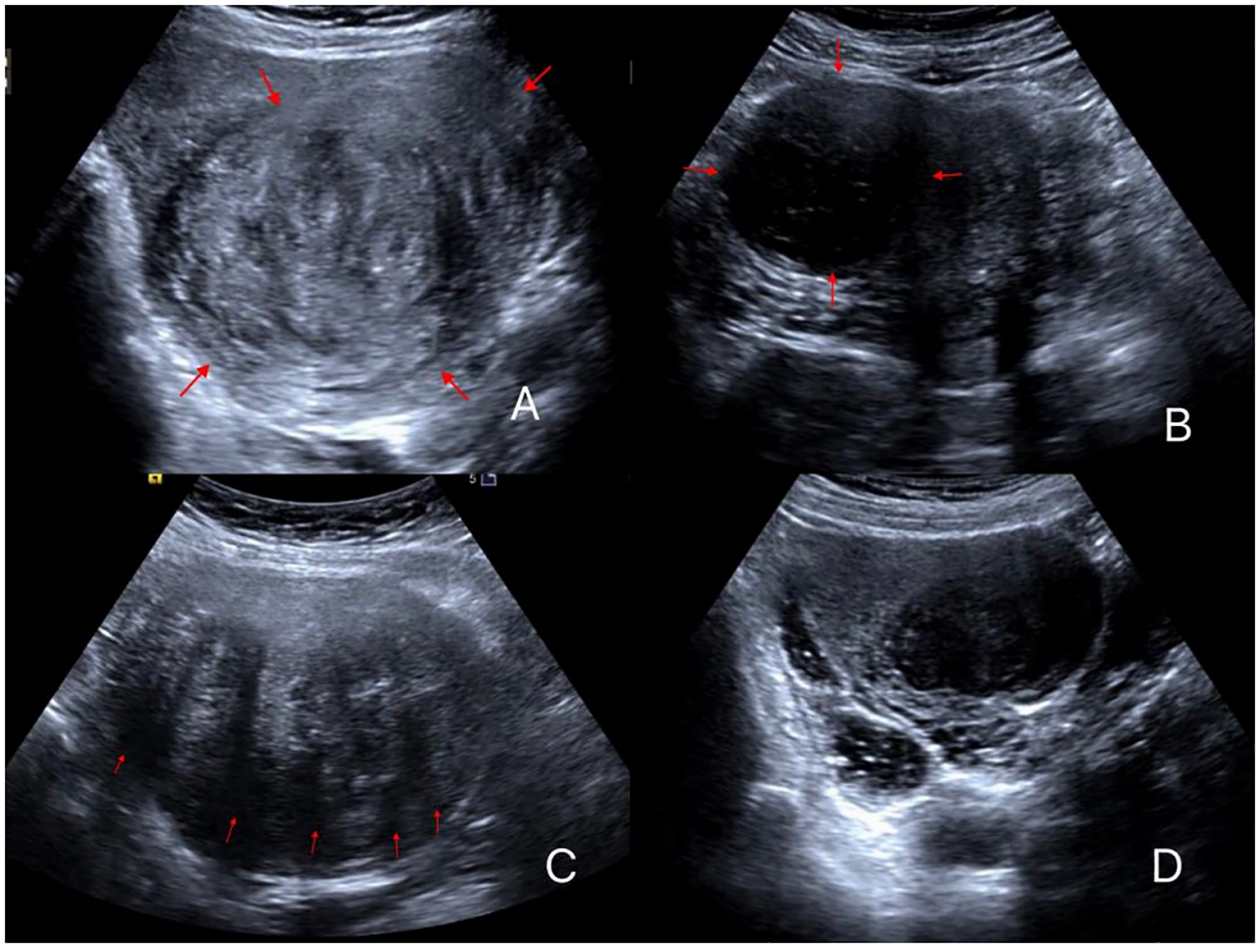
Different ultrasonic image features of uterine fibroids. **(A)** Fibroid is elliptical with high echogenicity; **(B)** Fibroid has a round shape with low echogenicity; **(C)** Fibroid with five attenuation bands; **(D)** Fibroids with no attenuation band and low echogenicity.

(1) Uterine fibroid location was classified into three types: anterior wall, lateral and fundal wall, or posterior wall.

(2) Uterine fibroids were classified into nine types using the International Federation of Gynecology and Obstetrics (FIGO) (11) classification system. Types 7 and 8 were excluded according to the exclusion criteria. The remaining seven types were grouped based on the location of the fibroids in the uterine wall. Types 0, 1, and 2 were classified as submucosal, types 3 and 4 as intramural, and types 5 and 6 as subserosal. Fibroids exhibiting characteristics of both types 2 and 5, protruding into the submucosa by ≤50% and the serosa by ≤50%, were classified as mixed-type fibroids.

(3) Uterine fibroid size was measured using three axes on maximum cross-sectional and vertical-sectional images. The volume of each fibroid was calculated using the formula V=0.5233×D1×D2×D3 (12).

(4) Uterine fibroids were categorized into two shapes, round or irregular (elliptical/diffuse or leaf-shaped).

(5) To assess the internal uterine fibroid echo, a comparison was made with the normal uterine muscular layer in the same horizontal plane, with consideration for abdominal wall thickness and interference from the intestines. Fibroids with internal echoes that were predominantly higher than the muscle layer were classified as hyperechoic, whereas those with lower internal echoes were classified as hypoechoic, and those with equal internal echoes were classified as isoechoic (**Figures 1A, B**). Fibroids with similar proportions of hypoechoic and isoechoic areas were grouped as hypo/isoechoic fibroids and the others as hyperechoic fibroids.

(6) The presence or absence of necrotic areas within the fibroids was determined based on the presence or absence of hypoechoic regions, thereby categorizing the fibroids into groups with or without necrotic areas, respectively.

(7) The number of posterior attenuation bands of the fibroid on the maximum cross-sectional image excluding lateral shadowing was counted (**Figures 1C, D**).

(8) Using the Adler grading system (13), blood flow was categorized into four levels: grade 0, grade 1, grade 2, and grade 3.

#### 2.4.3 Treatment method

Patients were placed in a prone position, and their vital signs and mental status were monitored. The patients underwent preoperative catheterization, bladder filling, and injection of midazolam (1 mg), sufentanil (10-40 μg), flurbiprofen ester (50 mg), and tropisetron (5 mg). During treatment, flurbiprofen ester (100 mg) and oxytocin (80U + 500 ml saline) were intravenously infused.

A preoperative scan was performed to formulate the HIFU ablation plan and adjust the focal point position. A layer-by-layer scanning approach was implemented throughout the ablation, sequentially progressing from point to line, line to plane, and plane to volume. This was done using a water cushion to displace the intestines. Ablation was conducted at a single point with a power of 400 W, applying continuous irradiation for 1 s, followed by a 1-s pause. Node spacing, pitch, irradiation time, and pause time ratios were adjusted based on real-time changes in the ultrasound images and patient responses. An immediate assessment using contrast-enhanced ultrasound was performed, and the absence of contrast agent perfusion indicated effective ablation.

After treatment, the patient’s conditions were observed, and the treated area of the skin and sensory-motor function in both lower limbs were examined. The patients were discharged after a 2-h rest period.

#### 2.4.4 MRI examination and observation parameters

The HIFU ablation rate was defined as the ratio of the volume of the ablated portion after HIFU treatment to the initial volume of the fibroid. It served as the primary indicator for evaluating the efficacy of HIFU, with a higher ablation rate indicating better effectiveness.

The energy efficiency factor (EEF) is the ultrasound energy required for HIFU ablation of 1 mm^3^ of tumor tissue. It acts as an index for measuring HIFU ablation efficiency and ease of ablation, with lower EEF values indicating higher ablation efficiency and easier ablation.

**Figure 2:**
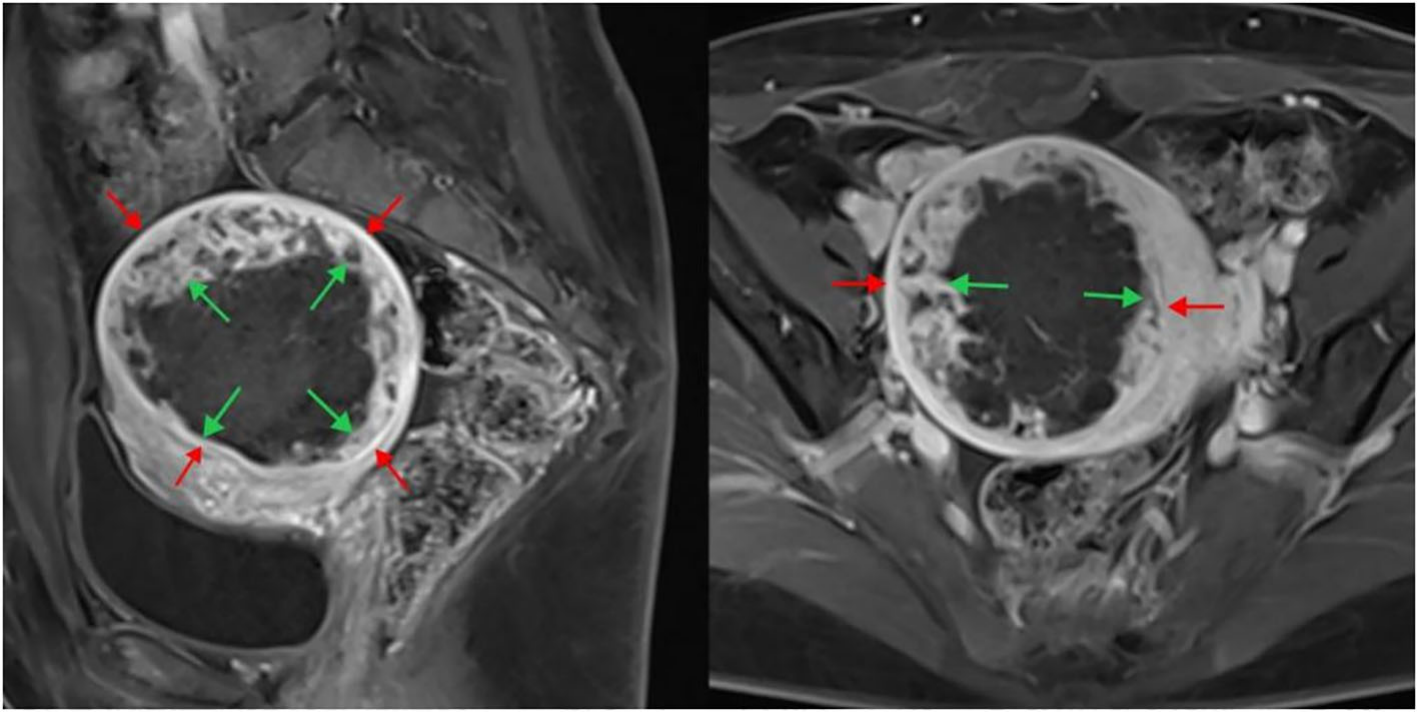
Enhanced MRI image after HIFU treatment of uterine fibroids. The sagittal plane is shown on the left, and the cross-section is shown on the right. The inner arrow indicates the size of the non-perfused area, and the outer arrow indicates the fibroid size. HIFU, high-intensity focused ultrasound; MRI, magnetic resonance imaging.


Contrast-enhanced MRI was conducted within 2 weeks before and after HIFU treatment. Routine spin echo (SE) sequence T1-weighted imaging (T1WI) and turbo spin echo (TSE) sequence T2-weighted imaging (T2WI) were performed in the cross-sectional, coronal, and sagittal planes, along with fat-suppressed sequences. The maximum cross-sectional images of the fibroids in the sagittal and transverse planes were analyzed postoperatively to measure the size of the targeted fibroids and non-perfused area. The volumes of the nonperfused area and fibroid were calculated in three dimensions (**Figure 2**): longitudinal (D1), anteroposterior (D2), and transverse (D3), using the formula V=0.5233×D1×D2×D3 (12). The ablation rate was computed as follows:

$$nonperfused\;volume\;ratio\;\left(NPVR\right)\;=\frac{nonperfused\;area\;volume}{targeted\;fibroid\;volume}\times \;100\%$$

Finally, the time taken for ablation was recorded to calculate the EEF using the following formula:

EEF = $\;\frac{\eta \times P\times t}{V}$ (J/mm^3^)

Where η is the focusing factor (=0.7), P is ultrasound power (W), t is the duration of ultrasound exposure, and V is the non-perfused volume (mm^3^) (14).

HIFU ablation is often performed in patients with multiple uterine fibroids; thus, non-target fibroids may partially absorb energy. To obtain accurate EEF data, we only calculated the EEF for single fibroids.

The sonographic features of the uterine fibroids, including position, classification, size, morphology, echogenicity, presence of internal necrotic areas, attenuation bands, and blood flow were recorded. The correlation between these features and the ablation rate, as well as the EEF of HIFU were analyzed.

### 2.5 Statistical analysis

All data were analyzed using IBM SPSS Statistics for Windows, version 26 (IBM Corp., Armonk, NY, USA). Categorical data are presented as frequencies, whereas continuous data are expressed as means ± standard deviations. Pearson’s correlation coefficient was used to assess the correlation between two continuous variables. The values ranged from -1 to +1, with higher values indicating a stronger relationship. Negative values denoted a negative correlation; positive values indicated a positive correlation, and zero values indicated no correlation. The significance level was set at *p*< 0.01.

Homogeneity of variance analysis was conducted to assess the overall distribution consistency for variables with count data and dependent variables with continuous data. If the homogeneity of variance was met, a one-way ANOVA (F-test) was performed to analyze whether the differences were statistically significant, assessing whether different levels of the same influencing factor impacted the dependent variable. If the homogeneity of variance was not met, Tamhane’s T2 test was used for multiple comparisons. Differences were considered statistically significant at *p*< 0.05. The results were illustrated through bar graphs and scatter plots with fitted curves for a more intuitive representation.

## 3 Results

A total of 75 patients with 124 uterine fibroids met the inclusion criteria and were included in the study. Among them, 41 patients had solitary fibroids, and 34 patients (83 fibroids) had multiple fibroids. Their ages ranged from 27 to 52 years, with a mean age of 43.5 ± 4.29 years. The maximum diameter of the fibroids ranged from 20 to 119 mm, with an average of 47.90 ± 19.54 mm.

### 3.1 Correlation between the sonographic features of uterine fibroids and the HIFU ablation rate

**Figure 3:**
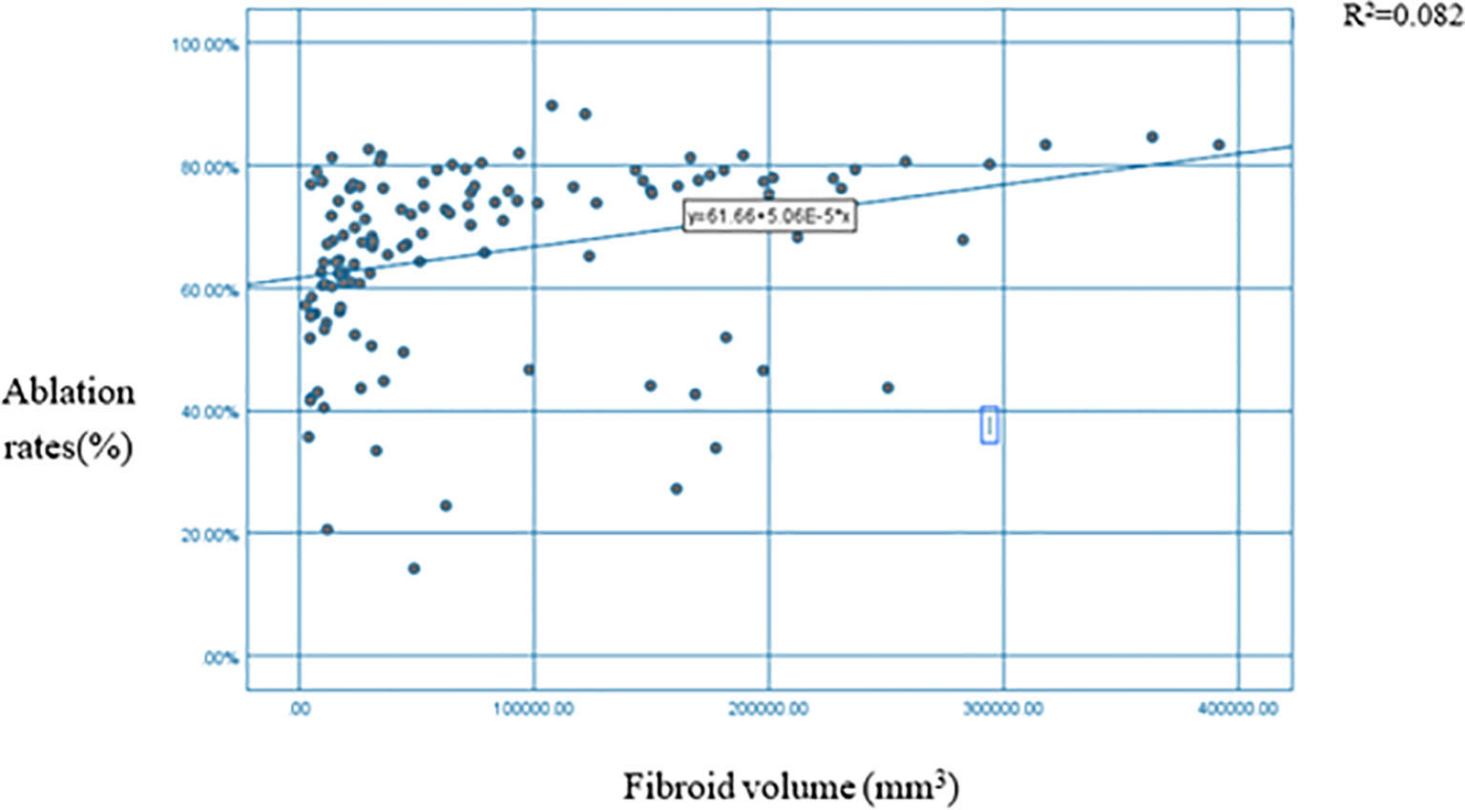
Scatter plot + fitting curve of myoma volume and HIFU NPV ratio. HIFU, high-intensity focused ultrasound; NPV, non-perfused volume.

The analysis revealed a significant positive correlation between uterine fibroid volume and ablation rate *(p* < 0.01) (**Table 1**). **Figure 3** illustrates a positive correlation between uterine fibroid volumes and ablation rates.

**Table 1:** Results of correlation between fibroid volume and HIFU NPV ratio.

Variable/Ablation rates (%)	Pearson Correlation (r)	P value
Fibroid Volume(mm^3^)	0.287	0.001

p< 0.01 indicates a significant linear relationship. HIFU, high-intensity focused ultrasound; NPV, non-perfused volume.

**Figure 4:**
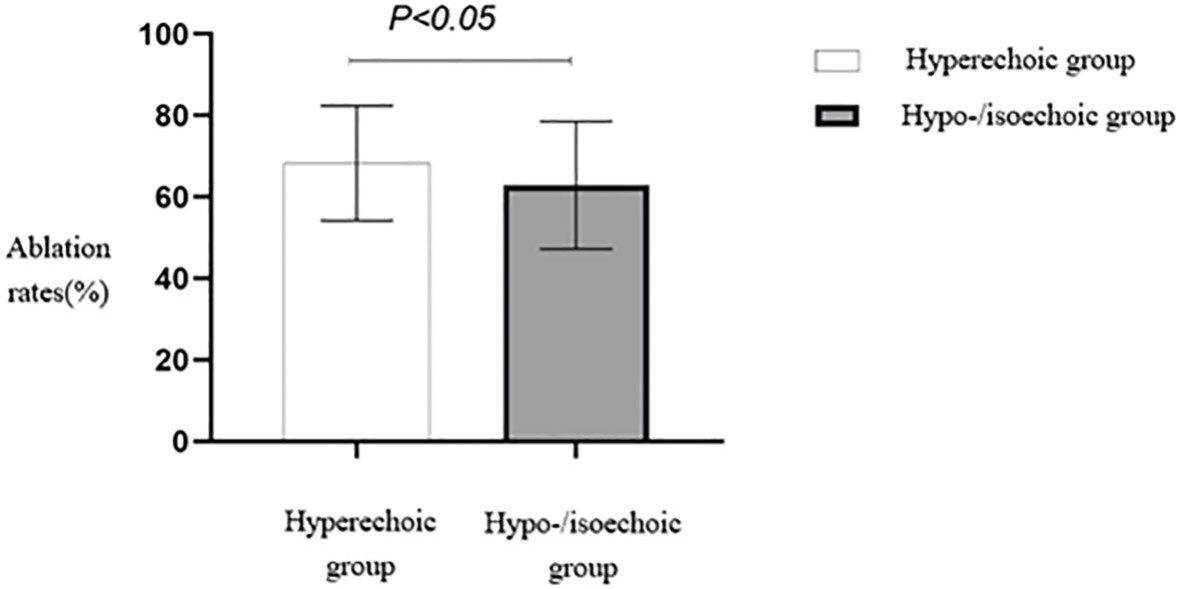
Statistical comparison of HIFU ablation rates of fibroids with different echo characteristics. HIFU, high-intensity focused ultrasound.

**Figure 5:**
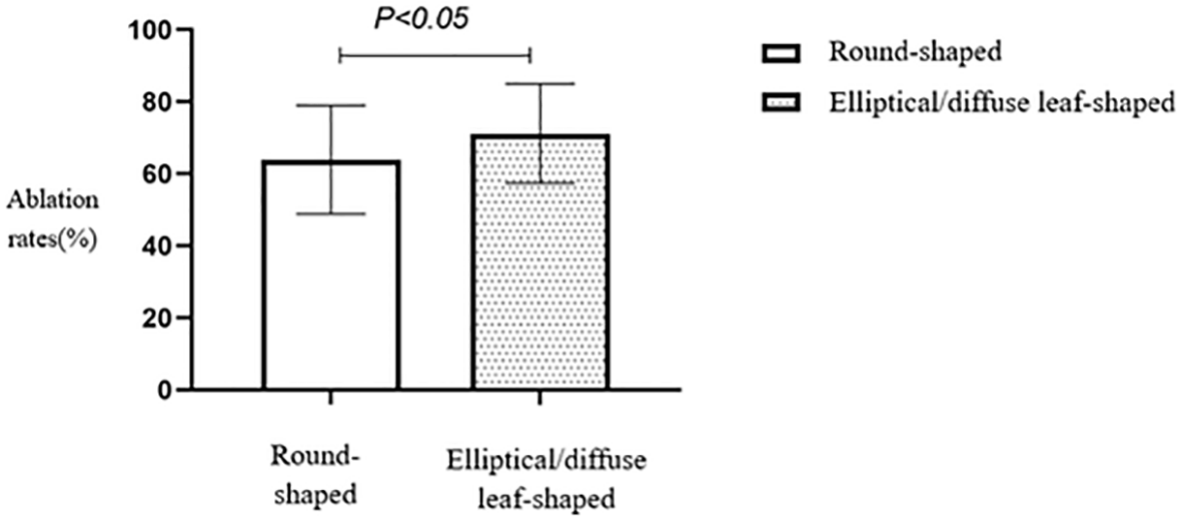
Statistical comparison of HIFU ablation rates for fibroids of different shapes. HIFU, high-intensity focused ultrasound.

The ablation rate in hyperechoic fibroids was higher than that in hypoechoic or isoechoic fibroids, and the difference between the two groups was statistically significant (*p* < 0.05) (**Table 2**, **Figure 4**). A comparison of HIFU ablation rates between the two groups based on morphology revealed that the elliptical/diffuse leaf-shaped fibroid group had a significantly higher HIFU ablation rate than the round fibroid group (*p* < 0.05) (**Table 2**, **Figure 5**).

**Table 2:** Correlation analysis between ultrasound features of fibroids and NPV ratio.

Ultrasound features of fibroids		Examples	Mean ablation rate(%)	F value	P value
Echo				4.145	0.044
	Hyperechoic fibroids	65	68.30 ± 14.07		
	Hypo-/isoechoic fibroids	59	62.87 ± 15.63		
Morphology				5.621	0.019
	Round-shaped	93	63.91 ± 15.04		
	Elliptical/diffuse leaf-shaped	31	71.15 ± 13.81		
Attenuation bands				6.296	0.013
	Absence of attenuation bands	63	62.46 ± 15.08		
	Presence of attenuation bands	61	69.09 ± 14.31		
Location				1.128	0.327
	Anterior wall	61	67.78 ± 12.16		
	Lateral and fundal wall	25	63.76 ± 17.76		
	Posterior wall	38	63.71 ± 17.08		
Type				2.379	0.073
	Submucosal	29	66.84 ± 13.90		
	Intramural	9	54.24 ± 16.39		
	Subserosal	41	68.42 ± 12.81		
	Mixed	45	64.83 ± 16.56		
Necrosis internally				0.139	0.710
	Absence of necrosis	118	65.83 ± 14.64		
	Presence of necrosis	6	63.49 ± 23.01		
Blood flow signal levels				2.510	0.086
	0	14	68.58 ± 15.53		
	1	45	61.78 ± 14.67		
	2	55	69.69 ± 10.97		
	3	10	57.64 ± 26.47		

Differences are statistically significant at p < 0.05. NPV: non-perfused volume.

**Figure 6:**
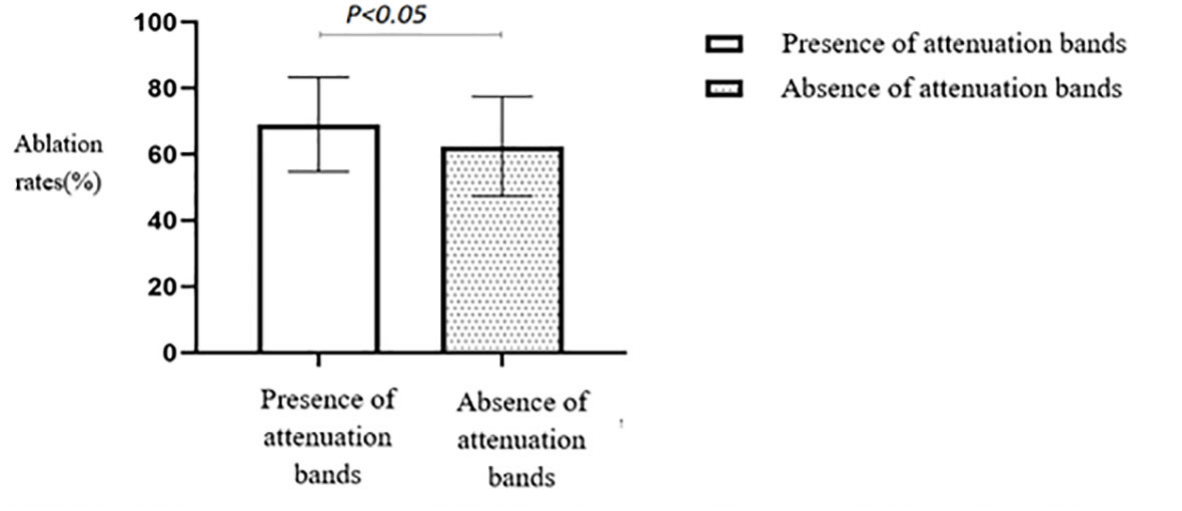
Statistical comparison of HIFU ablation rate with or without attenuated fibroids. HIFU, high-intensity focused ultrasound.

A comparison of the HIFU ablation rates based on the presence or absence of attenuation bands revealed that the group with attenuation bands had a significantly higher HIFU ablation rate than the group without attenuation bands (*p* < 0.05) (**Table 2**, **Figure 6**).

**Figure 7:**
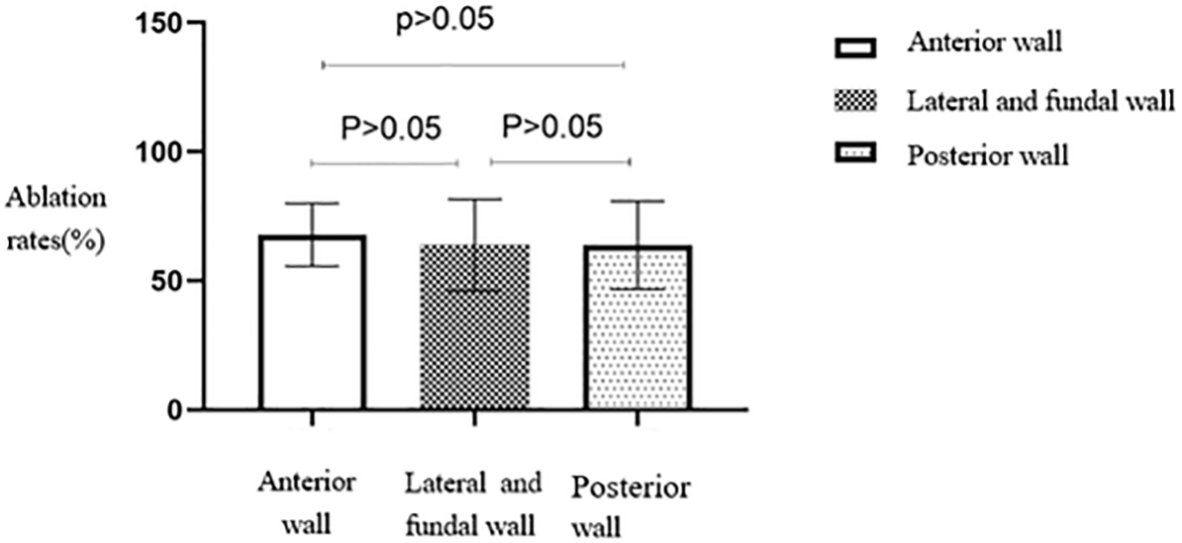
Statistical comparison of HIFU ablation rate of uterine fibroids at different locations. HIFU, high-intensity focused ultrasound.

No statistically significant differences were seen in the ablation rates among anterior, lateral and fundal, and posterior wall fibroids (*p* > 0.05) (**Table 2**). The anterior wall fibroid group had the highest average ablation rate, slightly higher than the average ablation rates of the posterior, lateral, and fundal wall fibroid groups (**Figure 7**).

**Figure 8:**
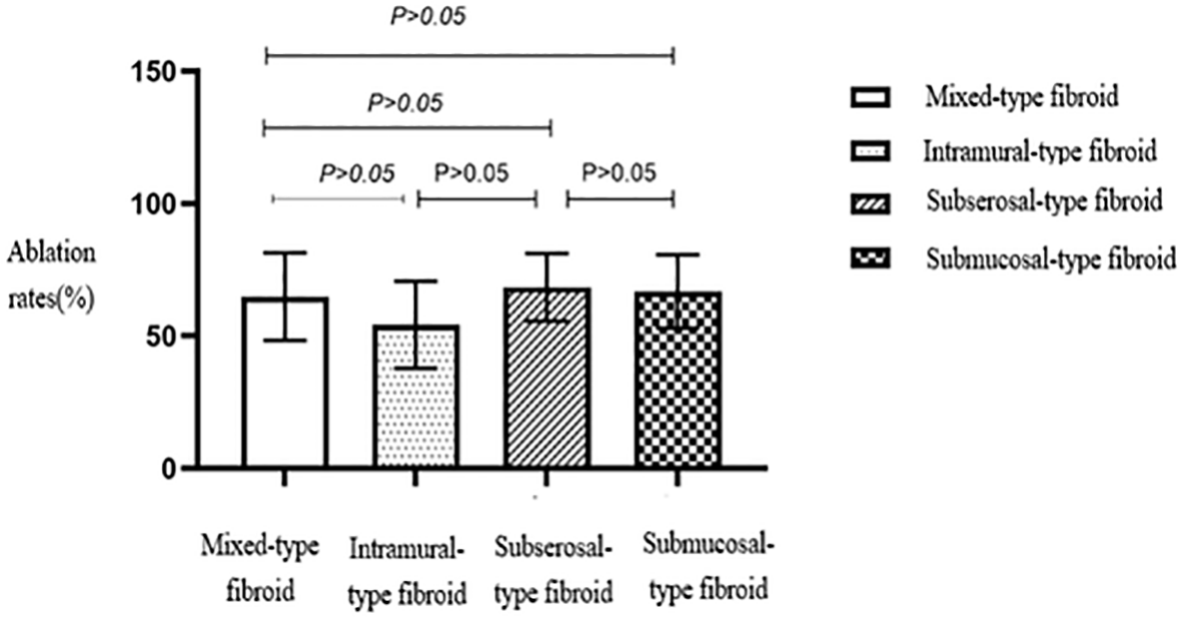
Statistical comparison of HIFU ablation rates for different types of uterine fibroids. HIFU, high-intensity focused ultrasound.

**Figure 9:**
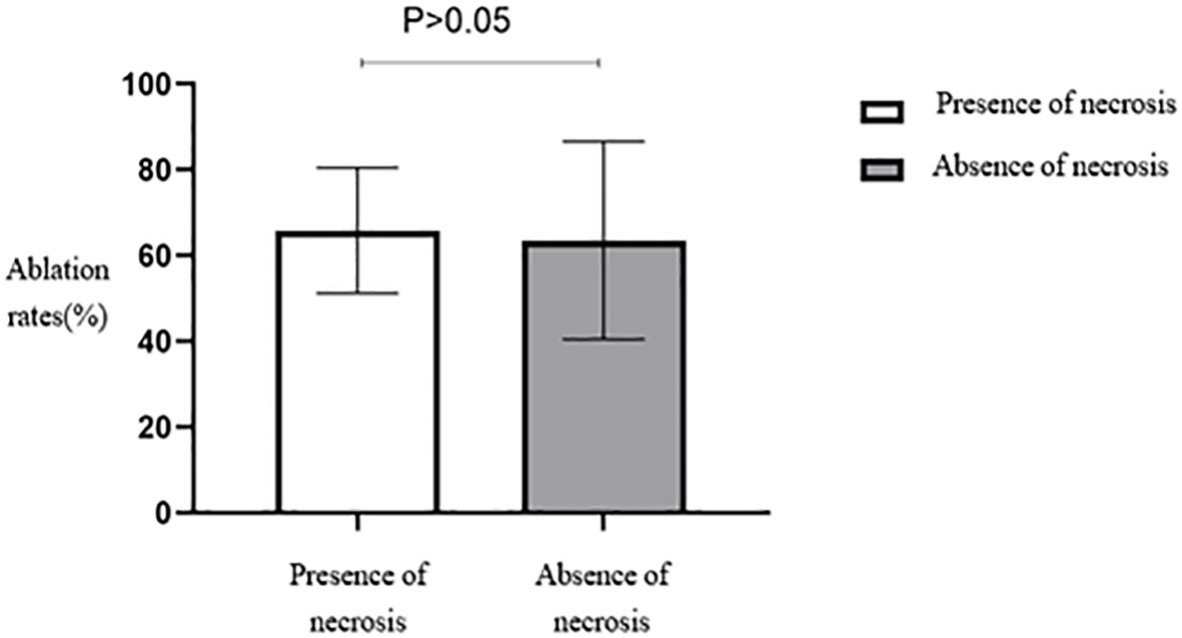
Statistical comparison of HIFU ablation rate of uterine fibroids with or without necrotic areas. HIFU, high-intensity focused ultrasound.

No statistically significant differences were seen in the ablation rates among the four different types of fibroids based on the FIGO classification (**Table 2**, **Figure 8**). Furthermore, no statistically significant differences were seen in the ablation rates between uterine fibroids without internal necrotic areas and those with necrotic areas (*p* > 0.05) (**Table 2**, **Figure 9**).

Analysis of the ablation rates among the four groups with different blood flow signal levels showed no statistically significant differences (*p* > 0.05). However, fibroids with a blood flow grade of 3 exhibited the lowest average ablation rate, significantly lower than those of the other groups (**Table 2**).

### 3.2 Correlation between sonographic features of uterine fibroids and the EEF

**Figure 10:**
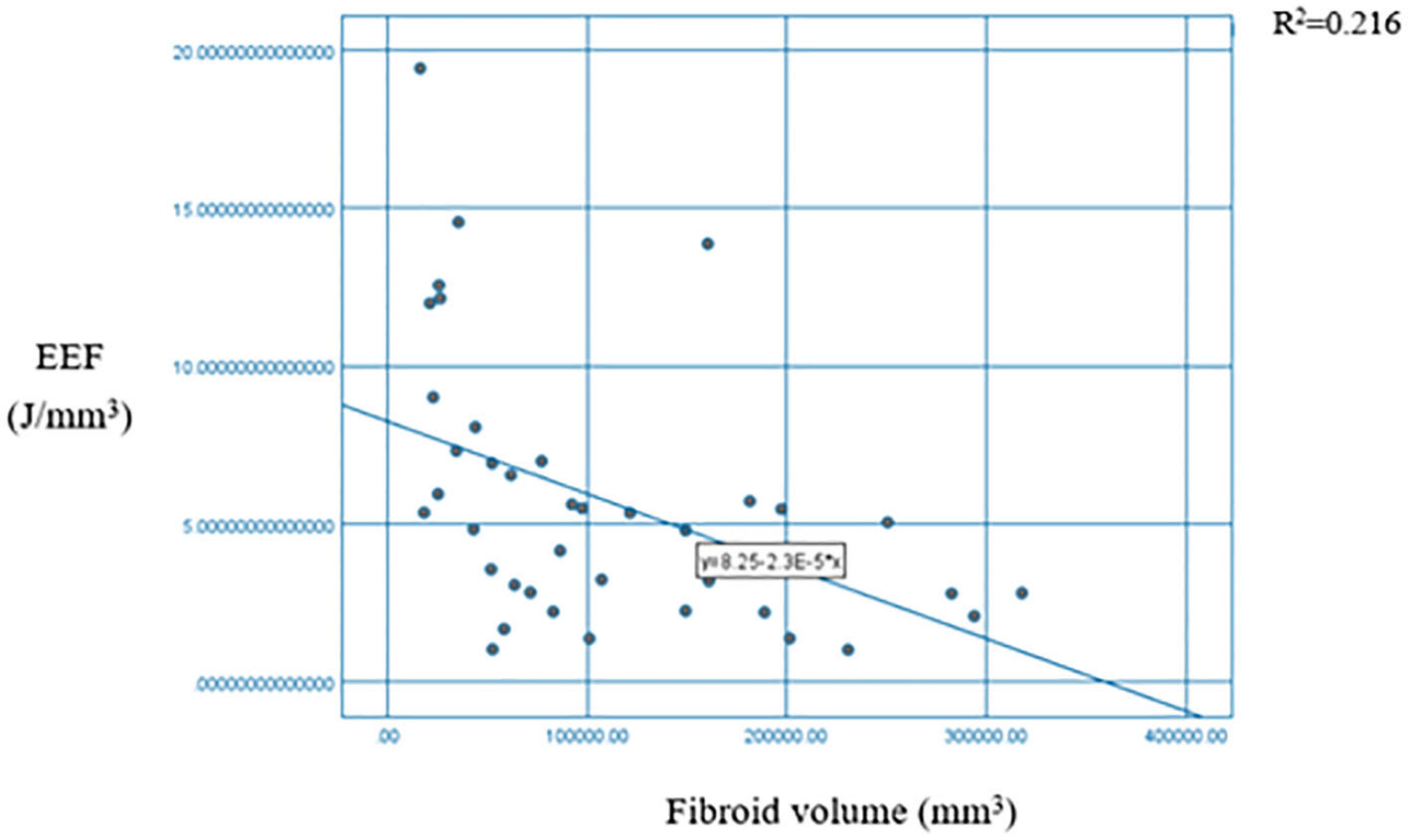
Scatter plot + fitting curve of fibroid volume and EEF. EEF, energy efficiency factor.

A significant negative correlation was observed between uterine fibroid volume and the EEF (*p* < 0.01) (**Table 3**). The scatter plot with a fitted curve also demonstrated an overall negative correlation, indicating that larger uterine fibroid volumes required lower ultrasound energy per unit volume for ablation (**Figure 10**), indicating a higher ablation efficiency.

**Table 3:** Results of correlation analysis between fibroid volume and EEF.

Variable/EEF	Pearson Correlation (p)	P value
Fibroid volume (mm^3^)	-0.464	0.002

EEF, energy efficiency factor.

**Figure 11:**
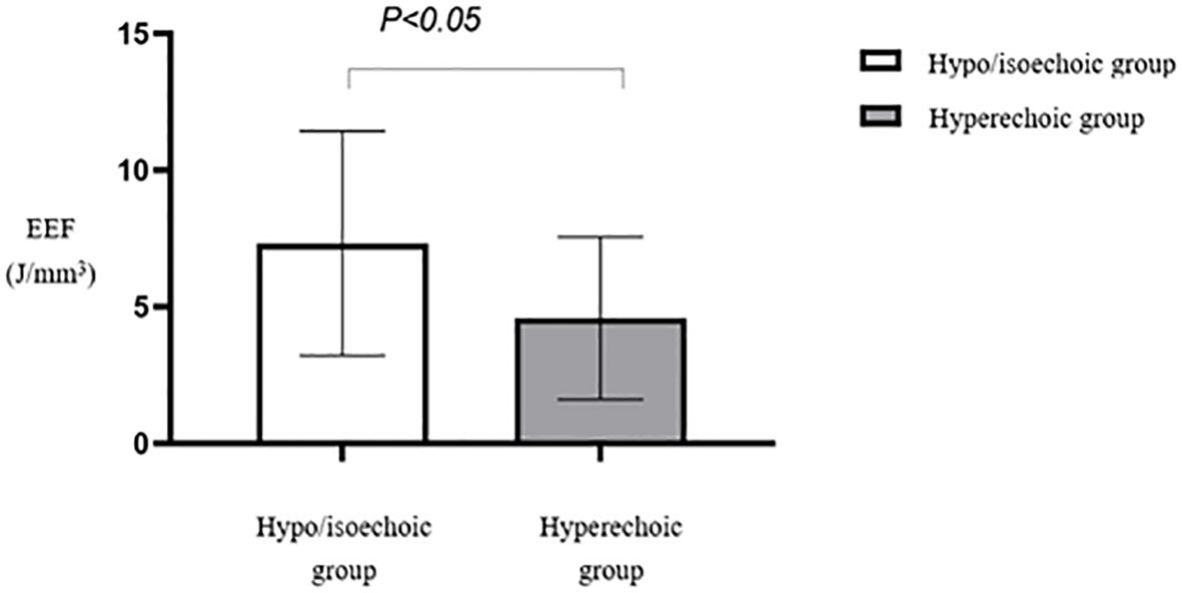
Statistical comparison of EEF of uterine fibroids with different echoes. EEF, energy efficiency factor.

Analysis of the EEF between low or equivalent echogenic uterine fibroids and high-echogenicity uterine fibroids revealed a statistically significant difference (*p* < 0.05). Fibroids in the low or equivalent-echogenicity group exhibited a higher EEF than those in the high-echogenicity group (**Table 4**, **Figure 11**).

**Table 4:** Correlation analysis results between different ultrasonic characteristics of uterine fibroids and EEF.

Ultrasound features of fibroids		EEF value (J/mm3)	F value	P value
Echo			5.535	0.024
	Hyperechoic fibroids	7.32 ± 4.11		
	Hypo/isoechoic fibroids	4.58 ± 2.97		
Necrosis internally			5.545	0.024
	Absence of necrosis	5.25 ± 3.80		
	Presence of necrosis	10.81 ± 5.90		
Location			0.350	0.705
	Anterior wall	4.02 ± 3.31		
	Lateral and fundal wall	4.26 ± 3.04		
	Posterior wall	4.60 ± 3.53		
Attenuation bands			0.769	0.386
	Absence of attenuation bands	6.45 ± 3.75		
	Presence of attenuation bands	5.25 ± 4.36		
Morphology			0.000	0.987
	Round-shaped	5.66 ± 3.99		
	Elliptical/diffuse leaf-shaped	5.64 ± 4.71		
Blood flow signal levels			0.463	0.711
	0	3.99 ± 0.80		
	1	5.83 ± 4.07		
	2	5.50 ± 4.34		
	3	7.64 ± 5.00		

Differences are statistically significant at p< 0.05. EEF, energy efficiency factor.

**Figure 12:**
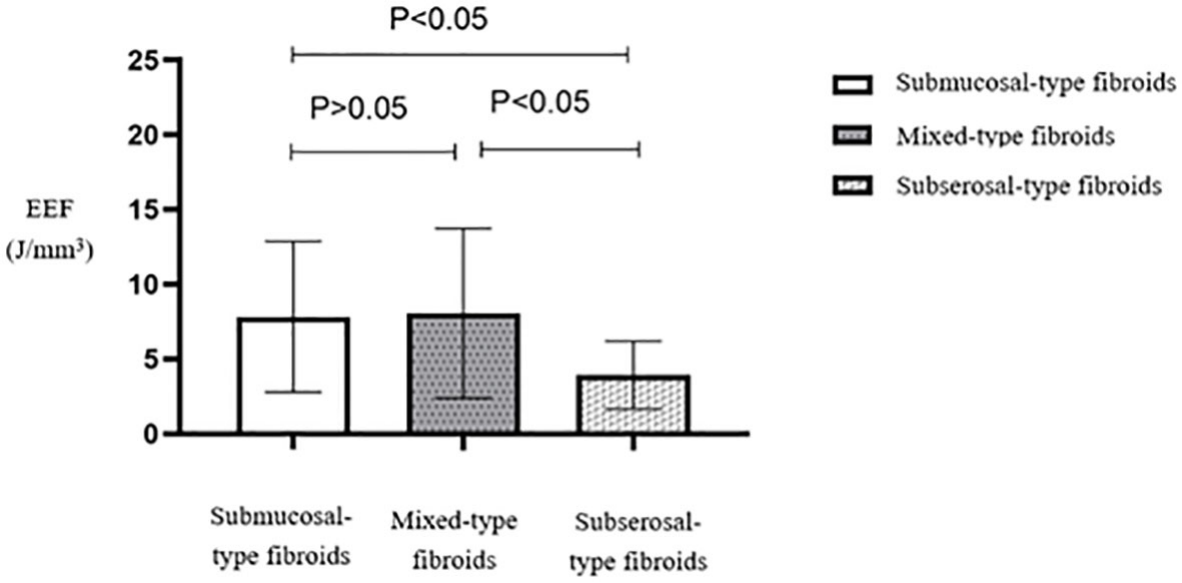
Statistical comparison of EEF of different types of uterine fibroids. EEF, energy efficiency factor.

**Figure 13:**
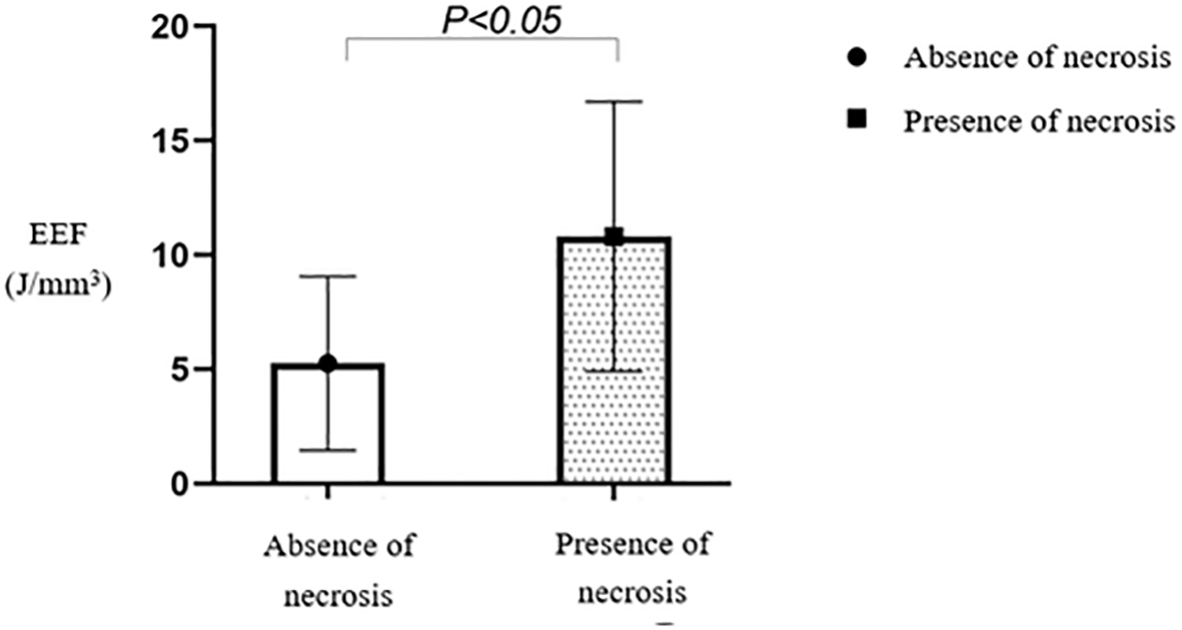
Statistical comparison of EEF in fibroids with or without necrotic areas. EEF, energy efficiency factor.

Multiple comparisons between fibroid types revealed statistically significant differences in EEF between subserosal and submucosal fibroids (*p* < 0.05) and between subserosal and mixed-type fibroids (*p* < 0.05). However, no statistically significant difference was observed between mixed-type and submucosal fibroids (**Table 5**). Subserosal fibroids exhibited a significantly lower EEF than submucosal and mixed-type fibroids (**Figure 12**). The EEF for fibroids without internal necrotic areas was lower than in those with internal necrotic areas (**Figure 13**), and this difference was statistically significant (*p* < 0.05) (**Table 4**).

**Table 5:** Multiple comparisons of EEF of different types of uterine fibroids.

Types of uterine fibroids/EEF	Subserosal fibroid	Mixed-type fibroid	Submucosal fibroid
EEF	3.93 ± 2.27	8.06 ± 5.68*	7.83 ± 5.04*

*Indicates a statistically significant difference compared to subserosal fibroids (p < 0.05).

EEF, energy efficiency factor.

**Figure 14:**
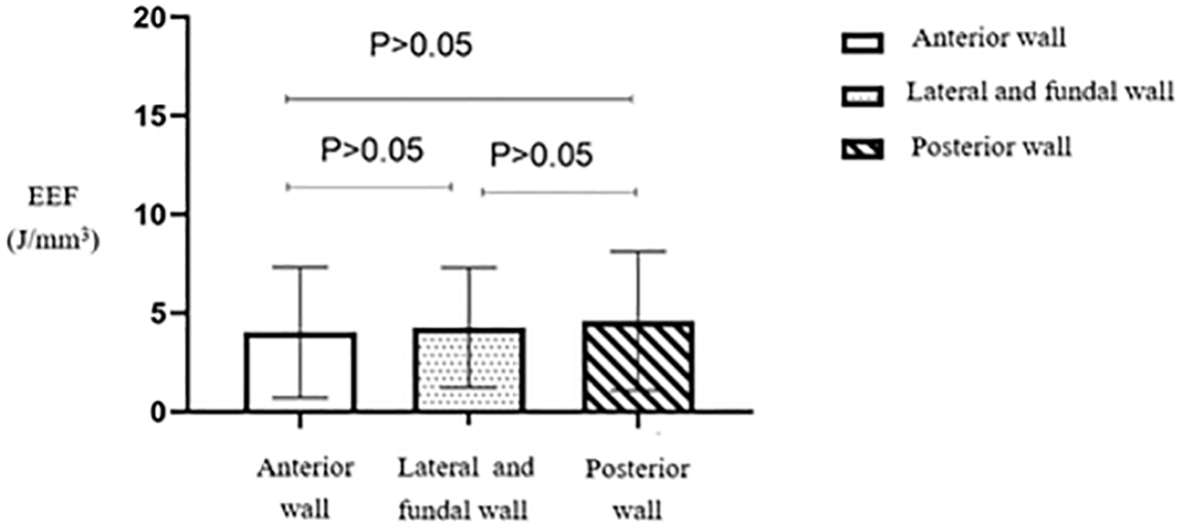
Statistical comparison of EEF of uterine fibroids at different locations. EEF, energy efficiency factor.

No statistically significant difference (*p* > 0.05) was observed in the EEF among the three groups based on fibroid location (**Table 4**). The average EEF value was lower in the anterior wall fibroid group, followed by the lateral and fundal wall fibroid groups. In contrast, the posterior wall fibroid group showed a relatively higher EEF (**Figure 14**).

The EEF comparison between the two groups based on the presence or absence of attenuation bands revealed no statistically significant differences (*p* > 0.05). However, the group with attenuation bands exhibited a lower average EEF than those without attenuation bands (**Table 4**). The EEF comparison between the groups with round and elliptical/diffuse leaf-shaped fibroids showed no statistically significant differences (*p* > 0.05) (**Table 4**).

The EEF differences among the four groups with different blood flow signal levels were not statistically significant (*p* > 0.05). However, the mean EEF in the group with a blood flow signal level of 3 was notably higher than that in the group with a blood flow signal level of 0 (**Table 4**).

## 4 Discussion

The study found a positive correlation between the volume of uterine fibroids and ablation rate and a negative correlation with EEF. This shows that larger fibroids were easier to ablate. *In vitro* experiments indicated that the EEF for ablating a segment of tissue was less than that for ablating linear tissue but more than the EEF for ablating a cubic tissue (15). This may be because larger fibroids occupy a larger acoustic pathway area, allowing more ultrasound energy to be delivered in the same amount of time while preventing cooling of the treatment area. The “damage-damage” interference effect may also explain this phenomenon. The dynamically changing area of necrotic tissue during ablation in the fibroid alters the “acoustic environment.” The high temperature reached during ablation changes the tissue properties (16), the destruction of blood vessels in the ablated tissue leads to a poor blood supply, indirectly preventing heat dissipation and thereby enhancing the ablation effect (17).

Our study observed higher ablation success in fibroids with greater echogenicity compared to those with lower echogenicity. This echogenicity disparity stems from variations in fibroid composition, such as the smooth muscle cells to fibrous tissue ratio, cellular attributes, and degenerative traits (18). Compared to fibroids with low or moderate echogenicity, those with high echogenicity present a significant difference in acoustic impedance between the components owing to their higher fibrous scaffold content and woven arrangement of smooth muscle cells (19). This results in increased ultrasound interfaces, making it more challenging for the ultrasound beam to penetrate, thus facilitating the deposition of ultrasound energy and consequently achieving better ablation outcomes.

We observed a difference in EEF values between fibroids with and without internal necrotic areas. Fibroids with necrotic areas exhibited higher EEF, suggesting that they may be more resistant to ablation. Increased free water content in fibroids undergoing degenerative necrosis facilitates ultrasound penetration but impedes energy disposition, affecting ablation outcomes. Additionally, a study revealed that tissues with a higher water content exhibited better thermal conductivity (20). Consequently, ultrasound energy deposition becomes more challenging, making ablation more difficult. Although no significant difference in ablation rates was found, prolonged procedure time and increased ultrasound dose may compensate for the resistance in fibroids without necrotic areas.

We noted a significant difference in ablation rates between fibroid groups exhibiting elliptical/diffuse leaf-shaped morphology and those with round morphology. The irregular elliptical/diffuse leaf-shaped fibroids displayed a higher ablation rate, possibly due to their larger size and intricate fibrous structure hindering ultrasound penetration. Consequently, ultrasound energy deposition was more effective, enhancing ablation efficacy. While the EEF did not significantly vary between different morphologies, the elliptical/diffuse leaf-shaped group exhibited a lower average EEF compared to the circular fibroid group, implying a higher susceptibility to ablation for fibroids with irregular shapes.

The presence of an attenuation band positively impacted ablation rates, attributed to the vortex-like structures, increased fibrous content, and denser smooth muscle cell arrangement associated with such fibroids. This led to enhanced ultrasound interface echo, facilitating energy deposition and subsequently higher ablation rates. Despite no statistically significant difference in EEF between groups with and without an attenuation band, the correlation between fibroid size, morphology, and ablation efficacy suggests that larger fibroids with irregular shapes or more attenuation bands are more amenable to HIFU treatment. Increasing sample size and employing controlled variable methods can provide more accurate insights into these relationships.

We observed no significant variation in HIFU ablation rates across different types of uterine fibroids, including submucosal, intramural, subserosal, and mixed-type fibroids. Clinicians tend to prolong the procedure time for a more thorough treatment of relatively resistant fibroids, thereby minimizing ablation rate differences. However, we noted a statistically significant difference in EEF between the subserosal fibroid group and the submucosal/mixed-type fibroid group. The subserosal fibroid group exhibited a notably lower average EEF, possibly due to their farther location from the endometrium and reduced amount of surrounding uterine tissue. This finding corresponds with Cheng Hailing’s observations (21) regarding the relationship between various MRI characteristics of uterine fibroids and HIFU efficacy.

In our study, anterior wall fibroids exhibited the highest ablation rates compared to posterior and lateral/fundal wall fibroids. This may be explained by minimal ultrasound attenuation as anterior wall fibroids are situated farther from the intestines and sacrum. Conversely, posterior wall fibroids are located near the sacrum and surrounded by abundant nerves and connective tissue, with severe ultrasound attenuation, making ablation more challenging (22, 23). However, this finding lacked statistical significance. It’s worth noting that certain studies have reported significantly higher ablation rates for anterior wall fibroids compared to lateral and posterior wall fibroids. These differences could stem from factors not accounted for, such as patient body mass index, subcutaneous fat thickness, body shape, and position, or limited sample size.

We found no significant correlation between ablation rates and the EEF in fibroids with different levels of blood flow. Some researchers suggest that fibroids with abundant blood flow may respond less to HIFU due to energy dispersion (22). Moreover, fibroids with more blood vessels are easier for ultrasound to penetrate, making energy deposition challenging. However, in our study, ablation efficacy did not notably vary based on blood flow conditions, likely due to the consistent use of oxytocin in all HIFU treatments. Oxytocin induces sensitive fibroid contraction, reduces perfusion, and mitigates blood flow’s impact during treatment (24). Nonetheless, our study’s limited sample size may have influenced these results, emphasizing the need for a larger sample size and controlled variables for more accurate future research.

This study has limitations as a retrospective analysis, where confounding factors like patient weight, subcutaneous fat thickness, and body shape may have impacted outcomes differently. Furthermore, variations in treatment efficacy could arise from HIFU procedures being performed by different physicians.

## 5 Conclusion

A higher HIFU ablation rate is associated with fibroids with larger volume, hyperechoic appearance, elliptical/diffuse leaf-shaped, and the presence of a posterior attenuation zone. A higher HIFU ablation efficiency is associated with fibroids with larger volume, hyperechoic appearance, subserosal location, and absence of internal necrotic areas. This study confirms that ultrasound imaging characteristics associated with uterine fibroids can predict the ablation rate and efficiency of HIFU treatment. It provides essential guidance for clinically assessing and selecting suitable cases for HIFU therapy, further assisting in the judicious selection of energy levels for treatment. Future research should explore the correlation between the ablation efficacy among different fibroid types and their origin from distinct clone cells. Furthermore, a large-scale multicenter prospective study with a standardized protocol and a larger sample size is warranted to confirm these findings.

## Data Availability

The original contributions presented in the study are included in the article/supplementary material. Further inquiries can be directed to the corresponding author.
